# Evidence for parallel adaptation to climate across the natural range of *Arabidopsis thaliana*

**DOI:** 10.1002/ece3.622

**Published:** 2013-06-07

**Authors:** Frank W Stearns, Charles B Fenster

**Affiliations:** Department of Biology, Biology-Psychology Building, University of MarylandCollege Park, Maryland, 20742

**Keywords:** Adaptation, arabidopsis, climate, parallel evolution

## Abstract

How organisms adapt to different climate habitats is a key question in evolutionary ecology and biological conservation. Species distributions are often determined by climate suitability. Consequently, the anthropogenic impact on earth's climate is of key concern to conservation efforts because of our relatively poor understanding of the ability of populations to track and evolve to climate change. Here, we investigate the ability of *Arabidopsis thaliana* to occupy climate space by quantifying the extent to which different climate regimes are accessible to different *A. thaliana* genotypes using publicly available data from a large-scale genotyping project and from a worldwide climate database. The genetic distance calculated from 149 single-nucleotide polymorphisms (SNPs) among 60 lineages of *A. thaliana* was compared to the corresponding climate distance among collection localities calculated from nine different climatic factors. *A. thaliana* was found to be highly labile when adapting to novel climate space, suggesting that populations may experience few constraints when adapting to changing climates. Our results also provide evidence of a parallel or convergent evolution on the molecular level supporting recent generalizations regarding the genetics of adaptation.

## Introduction

Climate is one of the most important factors determining the distribution of plants (Walther 2003) and therefore adaptation to climate should be a major selective force. Furthermore, the ability to adapt to climate heterogeneity can facilitate or constrain the dispersal of organisms, affecting species range (Angert et al. 2011), and climate adaptation may even play an important role in speciation (Keller and Seehausen 2012). Although historically local climates have been known to fluctuate across space and time at an ecological scale, human impacts are accelerating climate change and this has already affected the survival and distribution of some organisms (Parmesan and Yohe 2003; Parmesan 2006). The effects of climate change are expected to increase in the future (Hancock et al. 2011). Thus, the ability to adapt to different climate regimes will likely be an important factor in the persistence of populations and species. This is especially true of plants, which are sessile and less able to disperse to more favorable climates as climate change occurs.

Of particular interest is how labile populations are with respect to climate adaptation. That is, how easily are they able to expand their range into novel climate space, and how readily are they able to respond to climate shifts in their own range? The ability to predict the evolutionary dynamics that will result from widespread climate change will inform both conservation efforts and basic evolutionary theory (Bradshaw and Holzapfel 2001; Olsen et al. 2004; Teplitsky et al. 2008; Kearney et al. 2009; Hoffman and Sgro 2011; Hansen et al. 2012).

Studies of the effect of climate on species ranges have a long history in plant ecology and evolution (see e.g., Darwin 1859, ch. 11). Furthermore, there is extensive evidence for ecotypic variation within species that contributes to climate adaptation (e.g., Clausen 1926; Clausen et al. 1947; Lowry and Willis 2010). Although plasticity does play a role (Nicotra et al. 2010), the overall picture is that there is a significant genetic contribution to climate adaptation.

Large, publicly available data sets provide a wealth of information for genetic studies. Climate data are also widely available. Given that climate is a significant selective pressure, when populations have resided in a locality for a considerable time (number of generations), it is reasonable to assume that they have adapted to the local conditions. Therefore, combining such large-scale data sets allows researchers to estimate adaptation to climate on a greater scale than would be possible using experimental methods (Banta et al. 2012).

The mouse-eared cress *Arabidopsis thaliana* is an ideal candidate for such a study. *A. thaliana* exhibits an annual life-history strategy with a cosmopolitan distribution across a wide range of habitat types. As a model organism for genetic studies, *A. thaliana* strains from many different climate regimes have been extensively genotyped (Shindo et al. 2007). Climate is known to be an important feature affecting fitness of *A. thaliana* (Wilczek et al. 2009; Fournier-Level et al. 2011). Climate regimes have been experimentally shown to predict performance under common garden conditions (Hoffmann et al. 2005; Rutter and Fenster 2007). Finally, climate has been shown to be an important factor limiting the distribution of *A. thaliana* (Hoffmann 2002). Although only a few loci contributing to climate adaptation have been well studied, the emerging picture is that climate adaptation in *A. thaliana* is affected by a vast network of genes affecting traits such as tolerance to temperature (Westerman 1971) and drought (McKay et al. 2003). Loci related to climate adaptation have been found to be widespread throughout the genome by a genome scan (Hancock et al. 2011) and a recent study found a correlation between climate and particular nonsynonymous substitutions at the genomic level (Lasky et al. 2012). Despite this, few studies have empirically examined adaptation to climate in natural *A. thaliana* populations due in large part to the difficulty in conducting field studies across a large sample of populations (but see Agren and Schemske 2012). When environmental factors can be correlated with fitness, relying on publicly available environmental and genetic data allows for more comprehensive studies.

Here, we quantify whether the genotype of an ecotype is a useful predictor of the climate habitat it occupies. On the basis of earlier studies (Wilczek et al. 2009; Lasky et al. 2012), we expect the relationship between genetic distance and climate distance to be positive. However, how strong shared evolutionary lineage determines the ability to invade climate space is key to our understanding the lability of populations to adapt to climate. If there is a weak relationship between genetic relatedness and occupied climate space then it would suggest that there are multiple ways that a lineage can adapt to a particular climate regime, indicating high lability in the ability of this organism to adapt to climate. This question is highly relevant given the current state of drastic anthropogenic climate change. If the relationship between climate space and genotype space is limited, then it bodes ill for organisms like plants that may be restricted in their ability to escape unsuitable habitat.

## Methods

We used a large genetic data set from *A. thaliana* and a worldwide climate database to examine the relationship between genetic relatedness and occupied climate space. To compile data on a substantial number of ecotypes and to generate a genetic distance matrix, we took advantage of publically available data from a large-scale genotyping study (Borevitz lab: http://www.naturalvariation.org/hapmap). The *A. thaliana* accessions that we used were taken from 853 lines characterized at 149 SNPs. It was important to have evidence that the accessions had experienced the local climate for long enough to adapt to their collection climate locality. Thus, we attempted to only use accessions that were collected from less anthropogenically disturbed habitats (i.e., not roadsides) typical of *A. thaliana*'s natural habitat where they were more likely to have a relatively long history, and consequently enough time to adapt to local climatic conditions. Such habitats include steep rocky slopes, open areas near forest (but not in understory), and open habitats with sandy or limited soil. We included these habitats as well as habitats that reflect some human disturbance including fallow fields, rocky walls, cemeteries with sandy soil, and etc. In response to reviewers' suggestions we added a further 67 accessions that from the habitat descriptions appeared to be from more anthropogenically disturbed sites including roadsides, tourist parks, fields under active cultivation, railway ballasts, and etc. (Appendix B). However, the vast majority of lines had no habitat data and were omitted immediately. Of the rest, several were collected outside of the native range of *A. thaliana* (e.g., in North America) and several more were not genotyped at the majority of the SNPs. In addition, we excluded such habitats as “Botanic Garden” or any university-associated sites, as those plants may represent escaped accessions adapted to other localities. This resulted in a data set consisting of 60 accessions (Appendix A) that derived from what we consider the least anthropogenically disturbed habitats and 67 more accessions from sites that might reflect higher anthropogenic disturbance (Appendix B).

To generate a climate distance matrix among the 60 and 67 accessions, we compiled climate data for each locality from a database consisting of nine different climate factors recorded every 10 degree minutes worldwide. The closest recorded point to the collection site of each *A. thaliana* line was used for this study. In some cases this created overlap in the site data for certain accessions. While this data set does not capture what may be important microclimate variation, it was the most precise data available to us. Given that previous studies have demonstrated a genetic contribution to climate adaptation, we believe that this will provide a conservative measure of the lability of genetic adaptation to climate, as genotypes from populations in the same climate regime would be expected to increase the correlation between genotype and habitat climate. Data for eight of the climate factors were collected monthly. These were precipitation (pre), number of wet days (wet), mean temperature (tmp), mean diurnal temperature range (dtr), relative humidity (reh), sunshine (sunp), ground frost (frs), and 10-m wind speed (wnd). The ninth was elevation and consisted of a single measure for each location. We included all available climate factors to avoid any a priori assumptions about which factors were most important. We ran additional analyses on a selected subset of the data (mean temperature from November to June and precipitation from June to August). These factors were selected using Banta et al. (2012) as a guide. This reduced the partial Mantel correlation slightly and to a nonsignificant degree. We therefore included all climate factors in the final analysis. The data are from New et al. (2002) and can be downloaded from Climate Research Unit website (http://www.cru.uea.ac.uk/cru/data/tmc.htm).

To compare climate distance to genetic distance we calculated distance matrices for both genotype (SNPs) and climate. The genetic distance matrix was calculated using DNADIST from the PHYLIP package (Felsenstein 1989) using the F84 substitution model. The climate distance matrix was calculated using PROC DISTANCE METHOD=DGOWERS in SAS 9.1.3 (SAS Institute 2004). This is Gower's environmental distance metric (Gower 1971).

To compare the genetic distance matrix to the climate distance matrix using tree-based methods, we estimated neighbor-joining trees for each distance matrix using the program NEIGHBOR in the PHYLIP package (Felsenstein 1989). We then calculated the Robinson–Foulds tree distance metric using TREEDIST from the PHYLIP package (Felsenstein 1989). This metric measures the dissimilarity among the overall topology of two or more unrooted trees (Robinson and Foulds 1981). Smaller numbers indicate higher similarity among topologies. The scale of the metric ranges from 0 (total concordance) to 2*n* − 6, where n is the number of terminal nodes. In the situation where we used the 60 accessions, then the maximum Robinson–Foulds index would be 2 (60) − 6 = 114. We then used the program topd/fMtS (Puigbo et al. 2007) to calculate a Robinson–Foulds metric among a set of randomized trees. This number is expected to reflect low concordance due to random topologies.

We expected that geographic distance could inflate the relationship between genetic and climate distance because closely related genotypes are expected to share geographic locales and hence similar climates (Beck et al. 2008). Thus, to remove the confounding influence of geographic proximity, we calculated an additional matrix of geographic great circle distance in R (R Development Core Team 2011). We used the VEGAN (Oksanen et al. 2011) package in R to calculate the partial Mantel correlation (Mantel 1967) between the genetic distance matrix and the climate distance matrix controlling for the geographic distance matrix for both the 60 least disturbed and 67 moderately disturbed accessions separately and together. Mantel and partial Mantel tests are commonly used in ecology to study the relationship between ecological factors and genetic distance (Smouse et al. 1986). VEGAN calculates three correlation measures: Pearson's product moment correlation, Spearman's rank correlation, and Kendall's rank correlation. All R scripts can be found in the online supporting information.

## Results

**Figure 1:**
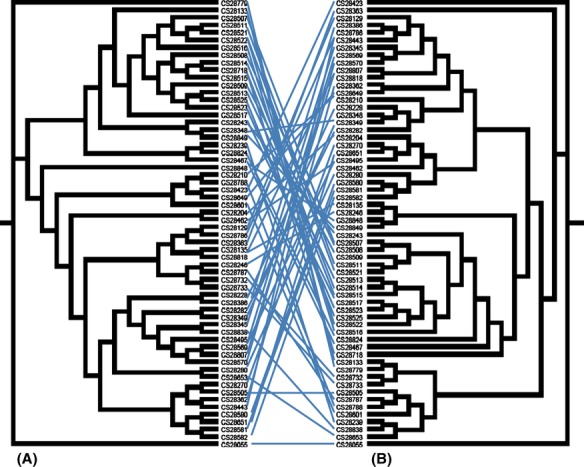
Neighbor-joining trees from NEIGHBOR in PHYLIP. (A) Tree reconstructed from a genetic distance matrix for *Arabidopsis thaliana* lines from across Europe and Asia. Pairwise genetic distance was calculated from 149 SNPs for 60 *A. thaliana* lines. (B) Tree reconstructed from climate data matrix for the habitat of each *A. thaliana* line. Pairwise climate Gower's distance was calculated from nine climate factors for 60 collection localities. Lines joining the two trees indicate which genotype (A) inhabits which climate space (B). There was overlap in collection sites for the 60 *A. thaliana* lines.

The genetic distance matrix (SNP) and the climate distance matrix for the 60 accessions collected from less disturbed sites are both represented as neighbor-joining trees (Fig. 1). The Robinson–Foulds distance metric for the two neighbor-joining trees was 110, indicating very low concordance between trees. The least possible concordance is 2*n* − 6, 114 in this case. The calculated Robinson–Foulds for a set of 100 randomized topologies for this data set is 113 with a 95% confidence interval of ±0.2. Therefore, although the concordance between these two trees is very low, there is a small signal of lineage on occupied climate space.

The results of the partial Mantel tests are presented in Table 1 for the 60 accessions collected from less disturbed sites that in our opinion more likely reflect native habitat. The partial Mantel correlations comparing the genetic distance matrix to the climate distance matrix and controlling for geographic distance were positive but low. Including the 67 moderately disturbed localities with the less disturbed (a total of 127 lineages) did not affect the ranked correlations from the partial Mantel test but reduced the Pearson correlation by about two thirds (from *r* = 0.23 to *r* = 0.07). When a partial Mantel test was conducted with the 67 lineages from the moderately disturbed localities alone, the Pearson correlation between genetic distance and climate distance was not significantly different from 0 (*r* = 0.02, *P* = 0.285). Therefore, we decided to base our conclusions only on the 60 accessions collected from less disturbed localities.

**Table 1:** The results of partial Mantel test estimating the correlation between genetic distance from 60 *Arabidopsis thaliana* lines and climate distance from their collection localities across Europe and Asia and controlling for geographic distance

	Partial mantel test	*P*
Pearson correlation (*r*)	0.2389	0.001
Spearman rank correlation (*ρ*)	0.07039	0.029
Kendall rank correlation (*τ*)	0.06944	0.003

Pairwise genetic distance was calculated from 149 SNPs. Pairwise climate Gower's distance was calculated from nine climate factors for each collection locality. There was overlap in some localities. The results are presented for Pearson correlation, Spearmen rank correlation, and Kendall rank correlation.

**Figure 2:**
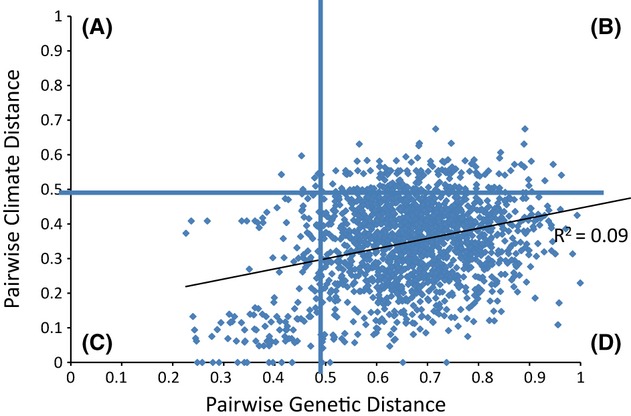
Scatter plot of pairwise climate data (Gower's distance) versus pairwise genetic data for 60 *Arabidopsis thaliana* accessions collected across its native range. As these data suffer from pseudoreplication, we did not include it in our formal analyses and only include it for illustrative purposes. The figure is divided into four quadrants representing general relationships between climate distance and genetic distance. They are (A) high climate distance and low genetic distance, (B) high climate distance and high genetic distance, (C) low climate distance and low genetic distance, and (D) low climate distance and low genetic distance. The majority of the points reflect a correlation between low climate distance and high genetic distance.

As an additional visualization we include a scatter plot of pairwise climate distance by pairwise genetic distance for the 60 accessions that shows a positive but low correlation, with the majority of the points reflecting high genetic distance coupled with low climate distance (Fig. 2). We acknowledge that pseudoreplication is a concern with this presentation and we do not base any of our formal analyses on this figure. It is included solely for illustrative purposes.

## Discussion

We demonstrate positive but low concordance between genetic relatedness in *A. thaliana* populations and the climate space that those populations inhabit. Both the partial Mantel tests and the Robertson–Foulds index indicate that genetic relatedness has little explanatory power in predicting the climate in which a genotype will be found. We interpret our results to mean that similar *A. thaliana* genotypes are able to occupy different climate regimes and that different genotypes have access or the ability to evolve to similar climate regimes. Therefore, access to different climate spaces appears to be relatively unconstrained by the *A. thaliana* genotype. This is also seen in Figure 2 where a number of genotypes have zero genetic distance based on the survey of 149 SNP's and yet occupy a wide range of climate regimes.

Hoffmann (2005) examined the evolution of climate adaptation in the genus *Arabidopsis* using phylogenetic reconstruction with climate space as a character. The analyses determined the core climate space (the climate space where all studied taxa coexist) and the realized climate niche (the intersection of taxa distribution ranges and climate data) of the genus. Hoffmann concluded that there was a high degree of parallel evolution to climate across the genus. Here, we demonstrate this same phenomenon within a species.

The ability of different genotypes to access similar climate habitats, and vice versa may help explain how *A. thaliana* has been able to achieve a cosmopolitan distribution in such a short period (e.g., across North America in approximately the last 150–200 years) (Vander Zwan et al. 2000; Jorgensen and Mauricio 2004). Given its annual life history it is possible that populations have adapted to climate on the order of tens to hundreds of generations. Recent range expansion in *A. thaliana* is certainly a result of human interference, but it is believed that the dispersal of self-fertilizing seed colonists has been the most important force in the history of the species. The ability of these annual colonists to rapidly adapt to novel climate habitats likely facilitated this process (Samis et al. 2012). Genetic adaptation to climate may exhibit the pattern we found due to parallel evolution or convergent evolution. In the former, the same genetic changes occur independently. In the latter, different genetic changes occur but the end result is the same. Several greenhouse and field studies have found a high beneficial mutation rate in *A. thaliana*, with as many as 50% of new mutations conferring a fitness advantage (Shaw et al. 2000; MacKenzie et al. 2005; Rutter et al. 2010, 2012). Thus, the independent adaptation to similar climates by genotypes that are not directly related may be due to the contribution of new beneficial mutations.

We foresee two potential concerns for our interpretation of independent adaptation to climate space. The first is the possibility that *A. thaliana* is phenotypically plastic with regard to climate space. Although this likely plays some role, we believe that our study also reflects genetic adaptation to climate. A reciprocal transplant study demonstrated genetic adaptation to climate between two European populations (Agren and Schemske 2012). Likewise, a common garden experiment has shown that the success of an accession of *A. thaliana* in a particular habitat can be accurately predicted by the similarity of that habitat to the native habitat of the ecotype (Rutter and Fenster 2007), consistent with adaptive differentiation to climate. Finally, analysis of the 67 accessions from localities we deemed that moderately disturbed showed no correlation between climate distance and genetic distance. We therefore feel our criteria for selecting localities were sufficiently conservative and reflect accessions that are likely to be locally adapted to their climate regime.

The second concern is that our study may not have captured variation in the loci that are involved in climate adaptation. The 149 SNP markers that we used to construct the genetic distance matrix were not intended to be used to identify loci associated with climate adaptation, although it is likely that some were given that linkage disequilibrium estimates vary from 10– 250 kb (Nordborg et al. 2002, 2005; Kim et al. 2007). Rather our SNP-based genetic distance tree clearly demonstrates that genetic relatedness is not a strong predictor of the climate inhabited by the genotype. We do know that climate adaptation in *A. thaliana* involves the interaction of a large number of loci distributed throughout its genome (Wilczek et al. 2009). In a recent study using 214,051 SNPs and 1003 accessions of *A. thaliana*, 15.7% of the genetic variation was found to be associated with climate (Lasky et al. 2012). Therefore, one would not expect all the same loci to be involved in the evolution to similar climates given the large number of loci so far identified to be associated with climate adaptation.

Based on our results, it seems likely that parallel evolution is common not just among species of *Arabidopsis* (Hoffmann 2005) but also within *A. thaliana*. An intriguing generality from adaptation genetics studies is that, at the sequence level, parallel evolution may be more common than once believed (Orr 2005a,b). If this is true, then species may not be as genetically constrained with regard to potential habitats, or adaptation to changing habitats. Our data indicate that *A. thaliana* is unconstrained with regard to climate adaptation within the range of climate in which it is found and this may account for its rapid cosmopolitan range expansion. Similar results were reported by Banta et al. (2012). These authors found that later flowering time restricted the niche breadth (measured by climate variables) of *A. thaliana* accessions as compared to earlier flowering accessions which were relatively unconstrained. Flowering time in their study could be controlled by any one of 12 different loci. That is, adaptation to climate could be affected by at least 12 different genetic pathways.

Our findings have important implications to conservation efforts that are responding to anthropogenic change (Bradshaw and Holzapfel 2001; Hoffman and Sgro 2011), suggesting that it may not be easy to predict which populations have the ability to adapt to new climate regimes. Using information from ecological and genetic databases in conjunction with smaller scale field studies may therefore be a useful way to generate results from a much larger number of populations than is feasible using experimental methods, and may help shed light on the genetics of climate adaptation.

## Appendix A

The 60 Arabidopsis thaliana accessions used in this study

**Table:**

Accession	City	Country	Latitude	Longitude	Habitat
CS28055	Bayreuth	Germany	49.941598	11.571146	Fallow land
CS28129	Calver	United Kingdom	53.269942	−1.642924	Rocky limestone slope
CS28133	Champex	Switzerland	46.313743	6.940206	Dry loam
CS28135	Chateaudun	France	48.069624	1.329393	Country road
CS28204	Dombachtal	Germany	50.091647	8.239811	Stony roadside
CS28210	Donsbach	Germany	50.722356	8.237199	Sunny, rocky soil
CS28228	Ellershausen	Germany	51.51052	9.682644	Limestone, south side
CS28239	Erlangen	Germany	49.599937	11.0063	Dry, sandy way
CS28243	Estland	Russia	58.595272	25.013607	Sandy hill
CS28246	Etraygues	France	44.644709	2.564473	Rocky slope
CS28270	Frankfurt	Germany	50.111512	8.680506	Fallow land, house garden
CS28280	Gieben	Germany	50.584007	8.678247	Edge of the forest
CS28282	Goettingen	Germany	51.532638	9.92816	Sunny slope
CS28345	Hohenlieth	Germany	54.268266	9.332247	Field
CS28348	Holtensen	Germany	51.809947	9.800169	Field
CS28349	Holtensen	Germany	51.809947	9.800169	Field
CS28362	Isenburg	Germany	51.834324	8.397892	Field
CS28364	Jena	Germany	50.926999	11.587011	Shaded new red sandstone
CS28386	Killean	United Kingdom	56.042425	−4.368315	Rocks on mica schist
CS28423	Krottensee	Germany	49.631206	11.572221	Rock outcrop
CS28443	Loch Ness	United Kingdom	57.322858	−4.424382	Rock ledges, moine schist
CS28462	Limburg	Germany	50.374069	8.122167	Fallow land
CS28467	Lipowiec	Poland	53.465086	21.136739	Loamy soil/Limestone
CS28495	Mainz	Germany	49.995123	8.267426	Sandy soil, cemetery
CS28505	Merzhausen	Germany	47.965954	7.828732	Roadside, stony loam
CS28507		Russia	61.27	34.56	Quarry
CS28508		Russia	61.27	34.56	Dry meadow on the rocks
CS28509		Russia	61.37	34.38	Rocks near the road
CS28511		Russia	61.83	34.4	Stool on the rocks
CS28513		Russia	61.88	34.55	Between two rocks
CS28514		Russia	61.97	34.58	Dry meadow on the rocks
CS28515		Russia	61.97	34.58	A small sandy hole
CS28516		Russia	61.97	34.2	Rock near ranger station
CS28517		Russia	62.02	34.12	Near the Lake Konchezero
CS28521		Russia	61.5	34	Mountain
CS28522		Russia	62.2	34.27	Rocks near town
CS28523		Russia	62.02	34.12	Rocks after the village
CS28525		Russia	62.02	34.12	Rocks near the road
CS28569	Noordwijk	Netherlands	52.234393	4.448311	Dune sand
CS28570	Noordwijk	Netherlands	52.234393	4.448311	Dune sand
CS28580	Oberursel	Germany	50.203323	8.576922	Sandy stony wall
CS28581	Oberursel	Germany	50.203323	8.576922	Sandy stony wall
CS28582	Oberursel	Germany	50.203323	8.576922	Sandy loam
CS28601	Pfrondorf	Germany	48.547803	9.110747	Sunny field
CS28649	Poppelsdorf	Germany	50.722039	7.088521	Sandy ground
CS28651	Praunheim	Germany	50.144782	8.607063	Loamy soil
CS28653	Poetrau	Germany	48.649	12.325969	Sandy fallow land
CS28718	Rubezhnoe	Ukraine	49.010799	38.381321	Near lake
CS28732	St.Georgen	Germany	48.122787	8.333986	Fallow land
CS28733	St.Georgen	Germany	48.122787	8.333986	Fallow land
CS28779	Tsagguns	Austria	47.077727	9.901948	Camping site
CS28786	Taynuilt	United Kingdom	56.428904	−5.239063	Rock ledges on basalt
CS28787	Umkirch	Germany	48.031687	7.761354	Embankment/River Dreisam
CS28788	Umkirch	Germany	48.031687	7.761354	Embankment/River Dreisam
CS28807	Wageningen	Netherlands	51.964641	5.662361	Papenpad in woods
CS28818	Weerseloo	Netherlands	51.358578	5.308936	Path in woods
CS28824	Wassilewskija	Russia	54.37773	19.433775	Sandy rye field
CS28838	Wu	Germany	49.632257	9.945612	Sandy soil
CS28848	Orsova	Rumania	44.714211	22.408039	Hill very close to Danube river
CS28849	Orsova	Romania	44.714211	22.408039	Hill very close to Danube river

The city and country where they were collected as well as the latitude and longitude are listed. Habitat data are also listed and were used in an attempt to restrict the study to less disturbed localities where local adaptation to climate has occurred.

## Appendix B

The 67 additional Arabidopsis thaliana accessions from moderately disturbed habitats

**Table:**

Accession	City	Country	Latitude	Longitude	Habitat
CS28007	Aun/Rhon	Germany	50.63544	10.11601	Field border
CS28009	Argentat	France	45.09336	1.93755	Railway ballast
CS28011	Achkarren/Frieberg	Germany	48.06781	7.62644	Vineyard
CS28013	Alston	United Kingdom	54.81217	−2.43868	Deserted garden
CS28014	Ameland	Netherlands	53.44056	5.65877	On dunes near firehouse
CS28049	Annecy	France	45.89925	6.12938	Garden
CS28050	Appeltern	Netherlands	51.83212	5.58465	Parking lot near show gardens
CS28051	Arby	Sweden	59.38194	16.52056	Country road
CS28053	Blackmount	United Kingdom	55.97291	−3.70978	Roadside 300 m
CS28054	Baarlo	Netherlands	51.32836	6.0876	Road side
CS28058	Buchen/Lauenberg	Germany	53.48325	10.61413	Deposited sand
CS28059	Buchen/Lauenberg	Germany	53.48325	10.61413	Deposited sand
CS28060	Buchen/Lauenberg	Germany	53.48325	10.61413	Deposited sand
CS28064	Bennekom	Netherlands	51.9991	5.67475	Roadside
CS28090	Bulhary	Czechoslovakia	48.83147	16.74874	Distr. Breclav (3 km E), left riverside of the Thaya, grassy place, outside wharf
CS28091	Boot, Eskdale	United Kingdom	54.39966	−3.27095	Parking lot pub
CS28100	Buchschlag/Frankfurt am Main	Germany	50.01475	8.70092	Near a rail line
CS28130	Canary Islands	Spain	28.29156	−16.62913	LasPalmas/Mirador
CS28134	Champex	Switzerland	46.02786	7.1165	Alpine garden
CS28216	Durham	United Kingdom	54.77525	−1.58485	Near cathedral
CS28217	Ede	Netherlands	52.04361	5.66667	Parking lot railway station
CS28234	Enkheim/Frankfurt	Germany	50.14222	8.75269	Field border
CS28240	Eringsboda	Sweden	56.43902	15.37709	In tourist park
CS28244	Estland	Russia	59.90436	23.78079	Railway slope near Pinsa
CS28269	Frankfurt/Niederrad	Germany	50.08833	8.64361	Roadside/River Main
CS28275	Gudow	Germany	53.55637	10.77171	Roadside
CS28279	Geleen	Netherlands	50.96912	5.82289	Park
CS28283	Goettingen	Germany	51.53835	9.92969	Near a highway
CS28344	Heythuysen	Netherlands	51.24748	5.90143	Garden
CS28347	Holtesen	Germany	51.56354	9.88978	Field of winter rye
CS28366	Jedburgh	United Kingdom	55.47772	−2.55494	Country road
CS28441	Lanark	United Kingdom	55.67386	−3.78214	Railway ballast
CS28453	Limburg	Germany	50.3986	8.07958	Near a lock
CS28454	Limburg	Germany	50.3986	8.07958	Near a lock
CS28455	Limburg	Germany	50.3986	8.07958	Near a lock
CS28457	Limburg	Germany	50.3986	8.07958	Roadside to Dietkirchen
CS28458	Limburg	Germany	50.3986	8.07958	Roadside to Dietkirchen
CS28459	Limburg	Germany	50.3986	8.07958	Railway embankment
CS28460	Limburg	Germany	50.3986	8.07958	Railway embankment
CS28461	Limburg	Germany	50.3986	8.07958	Thrown up earth
CS28466	Lindisfarne	United Kingdom	55.68077	−1.80086	Road side
CS28473	Le Mans	France	48.00611	0.19956	Wheat field
CS28490	Mickles Fell	United Kingdom	54.61572	−2.30183	Rocky ground on limestone
CS28510	Solomennoye	Russia	56.48347	31.6686	Road to Botanical Garden and soccer field
CS28518	Zarevichi	Russia	61.5	34	In the village near the chapel
CS28520	Konchezero	Russia	62.04679	34.11207	In the village
CS28524	Petrozavodsk	Russia	61.78886	34.35972	Segejskaya street
CS28563	Niederlauken	Germany	50.34472	8.43278	Roadside, loam
CS28572	Nieps/Salzwedel	Germany	52.69655	10.98148	Railway embankment, sand
CS28589	Otterburn	United Kingdom	55.23106	−2.17652	Parking lot shop
CS28638	Pitztal/Tirol	Austria	47.11667	10.78333	Roadside
CS28645	Pontivy	France	48.06615	−2.96706	Roadside
CS28667	Ravensglas	United Kingdom	54.35627	−3.40582	Parking lot railway station
CS28669	Ravenscar	United Kingdom	54.40191	−0.49084	Country road near pub
CS28672	Renkum	Netherlands	51.97609	5.73409	Garden
CS28685	Rhenen	Netherlands	51.96214	5.57112	Roadside
CS28691	Rome	Italy	41.90151	12.46077	Cerveteri monument
CS28692	Rouen	France	49.44323	1.09997	Roadside
CS28739	Siegen	Germany	50.88385	8.02096	Roadside to Hermesbach
CS28758	The Hague	Netherlands	52.0705	4.3007	Street near railway station
CS28759	Tingsryd	Sweden	56.52475	14.97853	Along main road in village
CS28760	Tivoli	Italy	41.95982	12.80223	Near ruins
CS28789	Umkirch	Germany	48.03446	7.76357	Sewage field
CS28800	Veenendaal	Netherlands	52.02344	5.55025	Parking lot railway station
CS28801	Veenendaal	Netherlands	52.02344	5.55025	Parking lot near house
CS28803	Vindolanda	United Kingdom	54.99224	−2.35527	Near Vindolanda restaurant
CS28808	Wageningen-Asserpark	Netherlands	51.96919	5.66539	Tree nusery near Asserpark
CS28819	Wilna/Litauen	Russia	54.68716	25.27965	Near Towniskaia
CS28821	Wilna/Litauen	Russia	54.68716	25.27965	Near Towniskaya
CS28850	Timisoara City	Romania	45.75554	21.2375	

Plane; continental and mediterranean climate. Roundhouse area of the main Timisoara railway station. Average annual temperature = 10.9°C. Average annual precipitation = 630 mm.

The city and country where they were collected as well as the latitude and longitude and habitat data are listed.

## Supporting Information

**Data S1.** R scripts.
