# Supplementary material for: Evidence for parallel adaptation to climate across the natural range of Arabidopsis thaliana
Source: Ecol Evol. 2013 Jun 7;3(7):2241–50. doi: 10.1002/ece3.622 (PMC3728961; doi:10.1002/ece3.622)
Supplement: Supplementary file 1 [file ece30003-2241-SD1.rtf]

R Scripts for Geographic Distance Matrix:
x <- read.table("infile.txt", header = TRUE) 
x.sp <- SpatialPoints(x) 
dist <- spDists(x.sp, longlat = TRUE) 
write.table(dist, "outfile.txt")

R Scripts for Mantel Test:
library(vegan) # load VEGAN package
x <- read.table("genetic_infile.txt", header = TRUE)
y <- read.table("climate_infile.txt", header = TRUE)
mantel(y, x, method="pearson", permutations=999)

R Scripts for Partial Mantel Test:
library(vegan) # load VEGAN package
x <- read.table("geographic_infile.txt", header = TRUE)
y <- read.table("genetic_infile.txt", header = TRUE)
z <- read.table("climate_infile.txt", header = TRUE)
mantel.partial(z, y, x, method = "pearson", permutations = 999)
